# Intensification of daily tropical precipitation extremes from more organized convection

**DOI:** 10.1126/sciadv.adj6801

**Published:** 2024-02-23

**Authors:** Jiawei Bao, Bjorn Stevens, Lukas Kluft, Caroline Muller

**Affiliations:** ^1^ Max Planck Institute for Meteorology, Bundesstrasse 53, Hamburg, 20146, Germany.; ^2^ Institute of Science and Technology Austria, Am Campus 1, Klosterneuburg, 3400, Austria.

## Abstract

Tropical precipitation extremes and their changes with surface warming are investigated using global storm resolving simulations and high-resolution observations. The simulations demonstrate that the mesoscale organization of convection, a process that cannot be physically represented by conventional global climate models, is important for the variations of tropical daily accumulated precipitation extremes. In both the simulations and observations, daily precipitation extremes increase in a more organized state, in association with larger, but less frequent, storms. Repeating the simulations for a warmer climate results in a robust increase in monthly-mean daily precipitation extremes. Higher precipitation percentiles have a greater sensitivity to convective organization, which is predicted to increase with warming. Without changes in organization, the strongest daily precipitation extremes over the tropical oceans increase at a rate close to Clausius-Clapeyron (CC) scaling. Thus, in a future warmer state with increased organization, the strongest daily precipitation extremes over oceans increase at a faster rate than CC scaling.

## Teaser

Daily tropical rainfall extremes increase when convective storms are more clustered.

## INTRODUCTION

Precipitation extremes are among the most damaging of natural hazards. Losses from precipitation extremes have been increasing. In July 2021, two flooding events triggered by heavy precipitation were estimated to be responsible for $54 billion in damages in Western Europe and $16.5 billion in damages in China (Munich Re NatCatSERVICE Natural catastrophes in 2021). Global warming is thought to be responsible for part of this increase (*1*). A consequence of the second law of thermodynamics is the exponential increase of the saturation vapor pressure of water with temperature, as described by the Clausius-Clapeyron (CC) relation (*2*). This means that for a given circulation and assuming a roughly constant relative humidity with warming, the water that is transported by this circulation increases accordingly with temperature, leading to the expectation that precipitation extremes will increase at a rate roughly matching that predicted by the CC equation. However, both observations and models show that precipitation extremes increase can strongly deviate from the CC scaling (*3*–*7*). While globally the rate of precipitation is constrained by the rate at which the atmosphere loses energy by radiative processes (*8*–*10*), locally, the rate of precipitation can vary by many orders of magnitudes relative to the mean.

Tropical clouds and convective systems are often spatially organized or clustered. They are manifested in a wide range of scales and exhibit diverse organization structure from small- and mesoscale systems such as squall lines and mesocale convective complexes to large- and planetary-scale systems such as tropical cyclones and Madden-Julian Oscillations. Precipitation extremes in the tropics are usually associated with these organized convective systems. In particular, observational studies either by tracking the mesoscale convective systems (*11*–*13*) or clustering cloud patterns using joint frequency distributions of the cloud top pressure and optical thickness from satellite images (*14*, *15*) have been attributing the occurrences of tropical precipitation extremes mainly to storms whose circulation features are associated with the meso-β (20 to 200 km). Changes in the behavior of convection on such scales, notably the degree of spatial clustering (here synonymous with organization) of convection, could be expected to impact the changes in tropical precipitation extremes. However, the scales of the processes involved are much smaller than can be represented by global models used for climate projections (typically 150 km) (*16*). Moreover, these models all use parameteric representations of convection, which are designed to represent the mean precipitation in a slowly varying environment, and which make no accounting for the geometric factors that define convective organization. This may explain why such models are unable to represent the types of precipitation systems responsible for extremes (*17*–*19*), and how they will change with warming.

In an effort to fill in this blind spot of the global modeling, researchers have begun using storm-resolving models (SRMs), which are distinguished by their use of a ca 3-km grid mesh to represent the transient dynamics of the precipitating convective systems responsible for the most extreme precipitation (*20*–*24*). Despite the model settings being idealized, important progress has been made in understanding the characteristics of convective storms, particularly how storms are initiated and maintained (*25*–*27*). Moreover, studying the convective storms arising from such simple configurations have led to the idea of measuring the degree of convective organization based on the spatial structure of organization (*28*–*30*), which in turn facilitates an enhanced understanding of organization and its impacts (*31*–*34*). Application of such idealized SRMs to investigate precipitation extremes suggests that daily precipitation extremes tend to increase substantially when mesoscale organization of convection becomes more clustered (*35*–*37*). However, a limitation of this approach has been that the simulations on which they are based are—for largely computational reasons—highly idealized. Typically, they allow the study of a single organized system under homogeneous large-scale forcing, in an inertial (nonrotating) double-periodic frame of reference. In reality, the organized convective systems, as introduced above, are more complex, as they are often modulated by the large-scale environment. Previous studies which measure convective organization in less idealized settings have been mostly satellite based analyses over regional domains, but links between convective organization and extreme precipitation can clearly be detected (*33*, *38*). It remains unclear whether a systematic relationship between convective organization and extreme precipitation by investigating the data across the broad tropics can still be found and how organization and its relationship with extreme precipitation will evolve in a warmer climate in the future.

As computational limitations have relaxed to the point where it has become practical to apply storm resolving models globally (GSRMs), to simulate less idealized conditions, and thereby better connect the dots between the idealized simulations and global climate modeling, many groups have begun exploring this new capability. The first intercomparison of GSRMs, DYAMOND, involved eight modeling centers from around the world to compare 40-day simulations of the atmosphere (*39*). Coupled versions of these models are also being constructed, and multi-annual simulations are being performed (*40*). This study uses simulations by the ICOsahedral Nonhydrostatic (ICON) model, both in a coupled and an atmosphere only configuration, for present, past, and future annual and semi-annual time slices, to understand how daily tropical precipitation extremes (total precipitation extremes accumulated over a day) are related to convective organization and to anticipate how both will change in response to warming. We compare coupled simulations of the year 2020 (2020, ICON_A/O_) with prescribed sea surface temperature (SST) time-slice simulations (year, ICON_A,year_). Three time-slices are compared, and one denoted by 2020 is based on SSTs from the European Centre for Medium-Range Weather Forecasts Integrated Forecasting System (IFS) analysis for the 2018–2020 period. Additional time-slices based on the SSTs associated with the years 1850 and 2070, whose mean tropical SSTs differ by about 2 K, are taken from a historical piControl simulation and from a warming simulation of the SSP585 scenario respectively both using the MPI-ESM 1.2-HR (*41*).

Daily variations in convective organization and extreme precipitation accumulations over the global tropics are compared to similar quantities derived from half-hourly precipitation maps as provided by the Integrated Multi-satellite Retrievals of Global precipitation (IMERG) dataset. Further details regarding the model and observations are provided in Materials and Methods.

## RESULTS

### Covariability of convective organization and daily precipitation extremes

We investigate the relationship between daily precipitation extremes and convective organization in the current climate by analyzing the statistics of deseasonalized measures of organization and extremes. Organization is measured by the clustering index (*I*_org_) which quantifies the spatial distribution of convective clusters (*29*). *I*_org_ is a nondimensionalized variable that measures the nearest-neighbor distances between convective clusters and then compares the cumulative density function of these nearest-neighbor distances against the distribution assuming that the clusters (with the same number) are randomly distributed. The values of *I*_org_ vary between 0 and 1 and are larger for more organized cases. We focus on the entire tropical domain (30°N to 30°S, land and ocean) and compute one value of daily precipitation extremes and one value of *I*_org_ for each day, thus obtaining daily time series of precipitation extremes and *I*_org_ (more details in Materials and Methods). Although we compute *I*_org_ over the entire tropical domain, the spatial scales that it captures are still small-, and mesoscales as *I*_org_ only considers the nearest-neighbor distances. This is confirmed by a recent study (*42*) as well as in fig. S1, which shows that most of the nearest-neighbor distances are shorter than 100 km, suggesting that the mesoscales dominate the organization behavior. To complement *I*_org_, we quantify the total number (*N*) and the average size (*S*) of convective clusters, as the convective clusters tend to be less numerous but larger in size with increased organization (*28*). Daily precipitation extremes are represented by percentiles (*n*th) of total precipitation accumulations over a day, *P_n_*. We use deseasonalized data (signified with δ) to avoid conflating signals arising from forced differences in the state of the large-scale circulation with changes due to an internal reorganization of the circulation. Hence, relative changes in organization or precipitation refer to changes that arise independently of seasonal forcing.

**Fig. 1.**
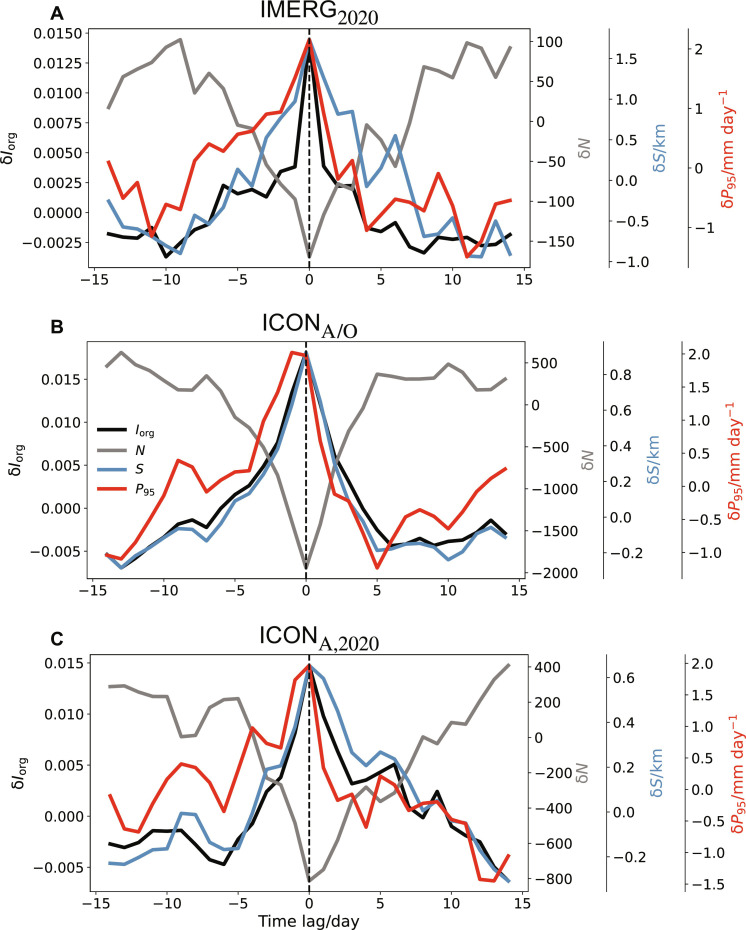
Time evolution of strong organization events. Time evolution of deseasonalized convective organization (δ*I*_org_) and 95th percentile of daily precipitation (δ*P*_95_), number (δ*N*), and size (δ*S*) during the composite peak organization events, from 14 days before to 14 days after the peak organization day. Peak organization days are the days when δ*I*_org_ > 90th quantile of δ*I*_org_ of the year and is larger than that on any other day in the previous and following 14 days. δ stands for deseasonalized data. The results are shown for IMERG_2020_ (**A**), ICON_A/O_ (**B**), and ICON_A,2020_ (**C**). Note change in scale.

We focus on the days when the degree of organization is relatively high (above 90th quantile of δ*I*_org_). In addition, to avoid overlapping in sampling, we restrict the analysis on the days when δ*I*_org_ is larger than that on any other day in the previous and following 2 weeks. This renders data samples of 31 4-week periods for IMERG, 27 4-week periods for ICON_A/O_, and 12 4-week periods for ICON_A_. We then analyze the mean time series of deseasonalized 95th percentile of daily precipitation (δ*P*_95_) and δ*I*_org_ composited over the 4-week period centered at the time of the maximum δ*I*_org_. As shown in Fig. 1, δ*P*_95_ and δ*I*_org_ tend to covary in both the coupled and uncoupled simulations. During such organization events, δ*I*_org_ varies by about 0.02 in the absolute value (2.5 to 3% in fractional change), and δ*P*_95_ varies by about 3 mm day^−1^ (8 to 10% in fractional change). Meanwhile, the size (δ*S*) and the number (δ*N*) are also closely related to δ*I*_org_, as δ*S* peaks on the same day when δ*I*_org_ maximizes, whereas δ*N* plunges to the lowest. Compared with the models, the time series of IMERG are noisier. Nonetheless the general trends of daily precipitation extremes and convective organization are consistent.

**Fig. 2.**
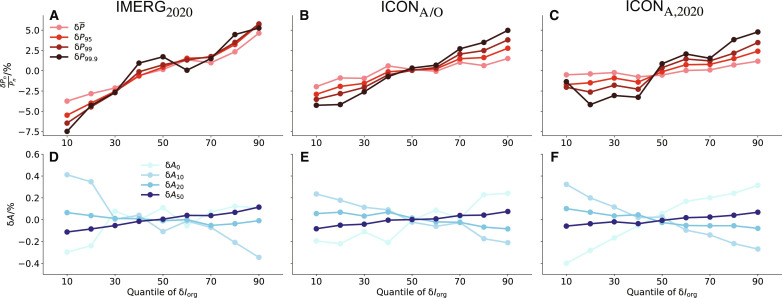
Precipitation amounts and precipitation area sorted by organization. The deseasonalized precipitation amounts [δ*P_n_* normalized by the mean *P_n_*, (**A** to **C**)] and the area of precipitation [δ*A*, (**D** to **F**)] as a function of mean quantiles of the deasonalized convective organization δ*I*_org_ (e.g., 50th quantile corresponds to the averaged values of δ*I*_org_ that is between 45 and 55th quantiles). The results are shown for IMERG_2020_ [(A) and (D)], ICON_A/O_ [(B) and (E)], and ICON_A,2020_ [(C) and (F)].

The close link between organization and daily precipitation extremes can also be seen in Fig. 2 (A to C) and fig. S2A in which δ*P*_*n*_ (normalized by the mean *P_n_* of the entire time period of each data) sorted by δ*I*_org_ increases systematically for both the simulations and the observation. Meanwhile, δ*N* decreases (therefore δ*S* increases) following the increase in δ*I*_org_ (fig. S3). The variations of δ*N* appear very small in IMERG, but the fractional change is of a similar magnitude as in the models (the much smaller *N* itself, which is explained later). We further quantify the link between organization and daily precipitation extremes by computing the correlation coefficients (*R*) from the deseasonalized daily time series (fig. S4A). *R* is calculated by conditioning on similar mean precipitation bins, as potentially δ*P*_*n*_ and δ*I*_org_ can be correlated because they are both correlated with $\delta \bar{P}$ through the influence of large-scale conditions. Conditioning the analysis on mean precipitation largely decorrelates δ*I*_org_ and $\delta \bar{P}$ but preserves the correlation with daily precipitation extremes. In both ICON_A/O_ and ICON_A_, the correlation coefficients are higher for δ*P*_95_ but tend to reduce for yet more extreme events. Observational results from IMERG qualitatively corroborate the above links between convective organization and daily precipitation extremes from ICON, but the correlations are weaker.

In the observations δ*P*_95_ and δ*I*_org_ appear to covary more nonlinearly (Fig. 1A). This might explain smaller linear correlations coefficients. Our analysis shows that in ICON the relationship between δ*I*_org_ and δ*P*_*n*_ is carried by storms that are on average smaller and more numerous (fig. S5)—associated with a tighter relationship carried by more events. In both ICON_A/O_ and ICON_A_, the average radius of a storm is about 20 km so that δ*S* is commensurately smaller and δ*N* is commensurately larger (Fig. 1). The reason why ICON simulates more, and smaller, storms, is not clear. This is not just an issue of ICON alone. Other kilometer-scale models with explicit convection have been reported to underestimate the size of convective storms (*43*–*45*). Candidate explanations would be the still relatively coarse (5 km) horizontal grid spacing, the representation of cloud microphysical processes, or perhaps their interplay, possibilities that seem amenable to tests by further simulations and by other groups.

Another explanation for a weaker linear correlation between δ*I*_org_ and δ*P*_*n*_ in the observations is that multicellular storms may be less readily distinguished by the observations. IMERG provides data on a 0.1° grid. However, the passive measurements on which it is partly based are not always so finely resolved over the global grid. Because the actual resolution of IMERG is coarser than the grid over which it is provided, this might lead to fewer but larger storms as compared to the simulations and weaken the observed relationship between organization and precipitation.

To explore this possibility, we investigate whether horizontal resolution plays a role in the above link between organization and daily precipitation extremes in the simulations. To do so, we regrid the model and observation data to coarser grids and repeat the analysis for δ*I*_org_ and δ*P*_95_. The correlations remain high even at 0.5° but reduce substantially at 1° (fig. S6). This suggests that the convective organization impacts are on 50- to 100-km scales, which are not so small as to explain differences between size and number of storms resolved by ICON versus by IMERG. The resolution dependence does, however, highlight that to represent organization and precipitation extremes for the daily variations in the current climate, models capable of representing yet finer, i.e., meso-γ (2 to 20 km) scale processes will be advantageous.

Not only daily extreme precipitation amount is intensified with more organized convection but also the whole distribution of precipitation changes (Fig. 2, D to F and fig. S2B). We quantify the total area of precipitation based on the absolute daily precipitation amounts. These include the dry area (*A*_0_: *P* < 1 mm day^−1^), the light-rain area (*A*_10_: *P* > 1 mm day^−1^ and *P* < 10 mm day^−1^), the moderate-rain area (*A*_20_: *P* > 10 mm day^−1^ and *P* < 20 mm day^−1^), and the heavy-rain area (*A*_50_: *P* > 50 mm day^−1^). Both models and observations show that the dry area (δ*A*_0_) and the heavy-rain area (δ*A*_50_) increase with δ*I*_org_, whereas the light-rain area (δ*A*_10_) and the moderate-rain area (δ*A*_20_) reduce. Thus, rain intensities become more inequitable with increased organization, and the situation of having either no rain or heavy rain becomes increasingly possible. This matters not only for extreme precipitation but also for extreme drying, which could compound the impact of phenomena such as heat waves or locally be associated with an enhanced probability of fire weather.

### Changes in convective organization and daily precipitation extremes with warming

As both models and observations show that the variations of daily precipitation extremes are related to the degree of mesoscale organization in the current climate, it becomes interesting to ask how daily precipitation extremes and organization will change in a warmer climate, and whether changes in precipitation extremes could be related to changes in organization.

**Fig. 3.**
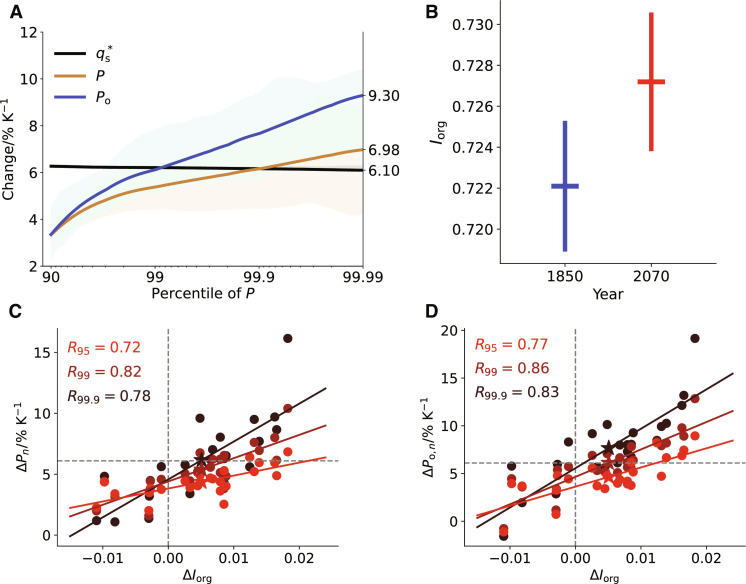
Changes in precipitation extremes and convective organization with warming. (**A**) Fractional changes in daily precipitation over all regions (*P*), over the ocean (*P*_o_) and surface saturation specific humidity ($q_{s}^{*}$) as a function of precipitation percentile. *P*, *P*_o_, and $q_{s}^{*}$ are sorted for each day and then averaged over each month from April to September. The changes are computed between 2070 and 1850 and are normalized by the mean surface temperature increase. The solid lines show the mean values of all months across four ensemble members. Shadings show the interquartile range. (**B**). Mean *I*_org_ in 1850 versus 2070. The error bars show SEs from monthly variations. (**C** and **D**) Changes in daily precipitation extremes over all regions [Δ*P*_*n*_, (C)] and daily precipitation extremes over the ocean [Δ*P*_o,*n*_, (D)] versus changes in organization (Δ*I*_org_). The changes are computed between 2070 and 1850 and are normalized by the mean surface temperature increase. Each dot represents the daily change averaged in 1 month, and colors from red to dark red indicate results for daily precipitation extremes at 95th, 99th, and 99.9th percentiles, respectively. Pentagrams are the mean values of all months. The horizontal dashed line in (C) and (D) is the corresponding changes in surface saturation specific humidity ($q_{s}^{*}$). The results are obtained from ICON_A_.

Figure 3A shows that daily precipitation extremes (*P_n_*) tend to intensify in a warmer climate. For the strongest extremes (99.99th percentile) over the whole tropics including both land and ocean, the ensemble-mean increases at a rate that is consistent with the surface moisture increase. However, substantial intensification is seen over the ocean (*P*_o,*n*_), at a rate of 9.3% K^−1^ for the 99.99th percentile, exceeding the changes in water vapor, which more closely follows CC scaling.

Could convective organization play a role in the intensification of daily precipitation extremes? First, we find that the degree of convective organization as indicated by the mean *I*_org_ over two periods tends to increase with warming (Fig. 3B). Estimates of the error in sampling the mean (whiskers) show that month-to-month variability is considerable; nonetheless, a signal can be clearly identified. The covariability between changes in daily precipitation extremes and changes in organization is evident and statistically significant (*P* < 0.005, 99.5% confidence interval) when comparing monthly realizations directly (Fig. 3, C and D): Months with a larger increase in organization have a larger increase in daily precipitation extremes with warming. Over the tropical oceans, without changes in organization, the rate of daily extreme precipitation increase is very close to CC scaling especially for *P*_o,99.9_. Increases in extremes are predicted to exceed CC scaling when convection becomes more organized. For precipitation extremes at higher percentiles, the intensification with increased organization is yet larger, as indicated by the steepening of the best fit regression lines: A small increase of 0.01 in *I*_org_ can lead to a further intensification of roughly 2% K^−1^ in *P*_o,95_ and 4% K^−1^ in *P*_o,99.9_ with warming. Unlike extreme precipitation, mean precipitation change is only weakly correlated with Δ*I*_org_ (fig. S7). This is expected as the mean precipitation change is tied to the net atmospheric radiation through the atmospheric energetic constraint (*9*, *10*), and the latter is not only affected by convective organization (*23*, *46*) but can also be sensitive to other processes (*47*).

To put the above relationship between the changes in monthly mean organization and daily precipitation extremes with warming (Fig. 3, C and D) in perspective, we compare the relationship arising from monthly variability within one climate which is not affected by warming. We focus on the data of 1850 alone and analyze the relationship between the variations of deseasonalized monthly mean *I*_org_ and *P_n_* in fig. S8 which only includes the signal from the monthly variability without the impact of warming. This yields correlations that are substantially lower, suggesting that the warming induced organization change further enhances daily precipitation extremes.

Figure 3A also implies that the ensemble-mean daily precipitation extremes over land increases at a sub-CC rate (less than 7% K^−1^). We do not find a robust relationship between changes in convective organization and changes in daily precipitation extremes over land. This may suggest that changes in precipitation extremes over land are more affected by large-scale processes as suggested by Pfahl *et al*. (*48*).

Besides precipitation extremes, changes in the dry area (Δ*A*_0_) are also positively correlated with changes in *I*_org_ (fig. S9A). Individual ensemble members all predict that the dry area will increase with warming, even in months when the degree of mesoscale organization decreases. Our interpretation is that the expansion of the dry areas is directly related to warming, and this signal is amplified by increased organization with warming. An increase in *I*_org_ is accompanied by decreased storm numbers but increased storm size, with both changes almost perfectly correlated with *I*_org_ (fig. S9, B and C).

### Changes in precipitation intensity versus duration

**Fig. 4.**
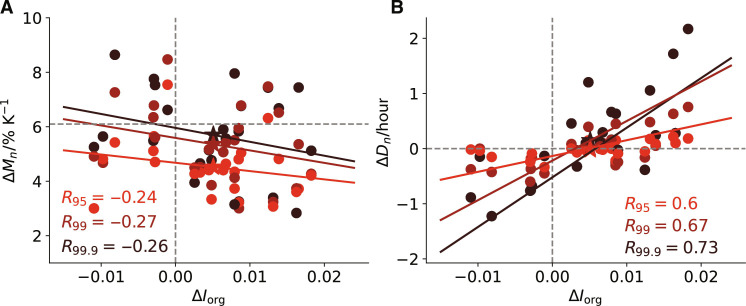
Changes in organization versus changes in intensity and duration of precipitation extremes. Changes in daily precipitation extremes (Δ*P*_*n*_) decomposed into changes in intensity [Δ*M*_*n*_, (**A**)] and duration [Δ*D*_*n*_, (**B**)] versus changes in organization (Δ*I*_org_). The details of the plots are the same as in Fig. 3C. The results are obtained from ICON_A_.

To further disentangle how convective organization affects precipitation extremes, we decompose daily precipitation extremes into precipitation intensity (*M_n_*) and duration (*D_n_*) (Fig. 4). Using the half-hourly precipitation data, we focus on the grids of *P_n_* and then count the total rainy hours within 1-day period. *D_n_* is the total rainy hours of the day, and *M_n_* is diagnosed by dividing *P_n_* by *D_n_*. The results show that it is primarily the changes in the duration of extreme events with warming that are responsible (statistically significant) for the close link between the changes in daily precipitation extremes and organization, while the intensity changes are weakly (not significant) and even negatively correlated with Δ*I*_org_. We speculate that the negative correlation (albeit weak) arises as a result of increased stability, leading to weaker instantaneous convective updrafts, in a more organized state. But this is counteracted by increases in the event duration, which are closely correlated with increased organization. The longer duration of daily extreme precipitation events can arise from the larger size of convective clusters with increased organization. Meanwhile, it could also be related to the slower storm propagation speed which, however, cannot be tested in this study. Regardless of the reason, our analysis suggests that organization affects precipitation extremes mainly by prolonging the event duration, consistent with what has been found in idealized studies (*35*, *36*). Here, we can show that, also in the case of more realistic model configurations, it is the event duration rather than extreme precipitation intensity that increases with more organized convection.

### Convective organization impacts the dynamics of precipitation extremes

Deviations from the CC scaling of the intensification of daily extreme precipitation with warming have been mainly attributed to the dynamics (or the storm kinematics) (*4*, *49*), which can come from changes in instantaneous convective updraft speed, or in its longevity in supporting precipitation over a particular area (precipitation duration). This raises the question whether the impact of convective organization on tropical daily precipitation extremes as proposed in our study contradicts the mechanism of dynamics? To understand this question and clarify the link between the dynamics and organization, first, we compare the time evolution of the deseasonalized daily precipitation extremes and the precipitation intensity, which is defined as the mean precipitation rate averaged only over the rainy hours of a day (fig. S10A). When the degree of mesoscale organization peaks at day 0, the daily extreme precipitation accumulations at 95th percentile (δ*P*_95_) also maximizes whereas the precipitation intensity (δ*M*_95_) minimizes. Consistent with Fig. 4, this suggests that in a more organized state, the increase of precipitation extremes accumulated over a day is caused by the increased duration with organization.

We further analyze the time evolution of dynamics composited by organization. The variations of the dynamics are represented by vertical velocity at 500 hPa (ω_500_). Focusing on the regions with daily precipitation extremes, the daily mean updraft velocity [ω_500_(*P*_95_)] is computed by averaging the hourly ω_500_ over the whole day (24 hours), while the hourly mean updraft velocity [ω_500_(*M*_95_)] is computed by averaging the hourly mean ω_500_ only over the rainy hours of a day (fig. S10B). The result shows that variability in the dynamics is always consistent with that in precipitation extremes: The daily mean updrafts [δω_500_(*P*_95_)] intensify when δ*I*_org_ peaks, while the hourly mean updrafts [δω_500_(*M*_95_)] decrease. The weakening in the hourly updrafts is due to the higher tropospheric stability with increased δ*I*_org_ (fig. S11).

An increase in convective organization leads to opposite responses in daily extreme precipitation amount and extreme precipitation intensity. It reduces the precipitation intensity by weakening the hourly convective updraft speed, but at the same time, an increased organization prolongs the precipitation duration which intensifies the dynamics for the total extreme precipitation accumulated over a day. Thus, we demonstrate that the changes in the dynamics of precipitation extremes are again modulated by the changes in convective organization.

## DISCUSSION

Tropical daily precipitation extremes are projected to intensify with increasing temperatures. However, it is unclear what process regulates tropical precipitation extremes and their change with warming, and this lack of understanding undermines confidence in the projections. Using a global SRM in a realistic configuration (both with coupled atmosphere-ocean simulation and with atmosphere-only simulations with prescribed SSTs), we show that the daily variations of tropical extreme precipitation accumulations and the mesoscale organization, as measured by the clustering index *I*_org_, are closely related in the current climate. Daily precipitation extremes tend to increase in a more organized convective state, accompanied by increased storm size but decreased storm number. This is broadly supported by observations, and the result is consistent with several recent observational studies, highlighting the impact of convective clustering on precipitation extremes over mesoscale domains (*33*, *50*). In a strongly warming scenario (SSP585), there is a robust increase in monthly-mean daily precipitation extremes. Over the tropical oceans, the highest precipitation percentiles (e.g., 99.9th or 99.99th) tend to increase faster than CC scaling. We find that such an intensification in tropical daily precipitation extremes is closely related to an increase in the degree of mesoscale organization through the impact of organization on the duration of the precipitation events. In general, an increase of 0.01 in *I*_org_ with warming can lead to a further intensification of roughly 2% K^−1^ in 95th percentile and 4% K^−1^ in 99.9th percentile of daily precipitation over the tropical oceans in addition to the rates without any change in *I*_org_ with warming.

The simulations demonstrate that, in general, the degree of mesoscale organization would increase in a warmer climate, with storms tending to get larger but less numerous. Increases in *I*_org_ also correlate with increases in the dry area, both in the current climate and future with warming. Thus, the precipitation intensity distribution becomes more inequitable. Such a shift of precipitation distribution to the two extremes implies more storms and more drying, even in the absence of mean precipitation changes. Enhanced future changes in wet and dry extremes have been reported by a regional SRM with explicit convection over Africa, but parameterized simulations with the same model show a weak or no signal (*51*). While GSRMs have been known to better simulate heavy precipitation processes (*19*, *44*, *45*), the representation of dry events may also be improved.

Our model predicts that, in general, the degree of organization would increase in a warmer climate, which, on one hand, could be forced by the changes in the large-scale processes, as individual convective systems and their spatial organization are often modulated by their large-scale environment. One study compared large-scale convective organization in CMIP5 models and found that 17 of 19 models show an increase in the large-scale convective organization with warming (*7*). The agreement among GCMs implies that the large-scale conditions may indeed favor organization. This is in contrast to the idealized SRM simulations across the RCEMIP ensemble in which there is no consensus on whether aggregation increases or decreases with warming (*23*). There are a number of reasons, many disputed, as to why the degree of the large-scale organization would increase with warming. For instance, the near-surface moist-static energy (peak values of which are associated with convection) increases faster with warming in regions with high sea-surface temperatures due to the exponential increase of water vapor with temperature. As the free-tropospheric temperature in the tropics is determined by the convective mixing of these regions of high near-surface moist-static energy through the troposphere, this would set a higher threshold for convective onset which disfavors convection in cooler and drier environments, thus facilitating organization (*52*, *53*). In addition, patterns of tropical sea-surface temperatures are expected to change with warming, in ways that may further favor the aggregation of convection (*54*, *55*). On the other hand, small-domain SRM simulations configured with idealized boundary conditions showed that small-scale processes such as cold pools (*56*) and turbulent mixing (*29*) could also play a role in convective organization, but their roles in a realistic context are not clear. One recent study used a different GSRM (NICAM) and forced the model with prescribed SSTs from reanalysis data for the current state and plus pseudo warming from CMIP3 models for the warming scenario. Although they found no indication of increased convective organization with warming (*57*), their model was configured with a much coarser resolution (14 km), and they did not measure organization directly from its spatial structure but rather inferred it from impacts of organization. Thus, the role of the small-scale processes in shaping organization remains to be identified and evaluated with more GSRMs which will likely be available in the near future. While it is difficult to understand what leads to an increased organization with warming, we would like to emphasize the key message of the study: Changes in tropical daily precipitation extremes are closely related to the changes of mesoscale organization of convection. Therefore, whatever process leads to an increase in convective organization, it can potentially intensify daily precipitation extremes.

The link between convective organization and daily precipitation extremes appears to be weaker in IMERG than in the models. Partially this is because the current resolution used in GSRMs is still relatively coarse in representing the convective processes (increasingly so for shallow storms). This results in more but smaller storms (*43*–*45*), which may contribute to a stronger relationship between changes in convective organization and daily precipitation extremes in the models than in observations. This may imply that the model tends to overexaggerate the importance of convective organization. On the other hand, precipitation from IMERG (especially over the oceans) is mainly based on the satellite retrievals. The passive measurements on which it is based are interpolated from infrared measurements to fill space and time gaps in the microwave. This, combined with issues such as beam filling and uncertainty in mapping the microwave signature to actual rainfall, would be expected to lead to a lower effective resolution than the grid over which it is provided. Despite that, the modeled relationship between convective organization and daily precipitation extremes is still stronger than the observed one even when the model data is regridded to 0.5°. Thus, more work is anticipated to investigate the discrepancies between GSRMs and observations in representing convective organization.

The above results about the changes in precipitation extremes and convective organization are based on one model simulation. Because of computational constraint, we cannot address the model uncertainty issues regarding the future projections. More GSRM simulations are anticipated in the future, through joint collaborations across different modeling centers, to better understand a range of key physical processes and tackle various climate issues including testing the robustness of our results here.

Our analysis, enabled by a new generation of global climate models designed to resolve the processes underlying changes in extreme precipitation, should motivate efforts to better understand changes in convective organization. The processes underpinning convective organization and the scales on which they happen are absent in the models heretofore used to study global climate change. For example, global climate models used in the last phase of the coupled model intercomparison project were based on coarser grids (typically 150 km), and (more importantly) all used parameteric representations of convective systems, designed to represent the mean precipitation in ways that do not account for convective organization and do not allow for the mesoscale circulation response found to underpin the changes in extremes in the present simulations. Global storm resolving models enable not only a more physical basis for the prediction of extremes but also the empiricism that will be required to advance understanding about future changes in precipitation extremes—all the more so as their resolution and duration will be refined in coming years.

## MATERIALS AND METHODS

ICON_A/O_: We use simulation output from global storm resolving simulations by ICON in a coupled (atmosphere and ocean) configuration. During the model development, three sets of 1-year simulation have been performed to fix the emergent issues in the model which accidentally creates different SST patterns [fig. S12, including observed SSTs from DOISST (*58*) for reference]. Each simulation has a distinct SST pattern and was run from 21 January to 31 December in 2020. These three sets of 1-year simulation do not differ qualitatively in their representation of convective clustering and extremes, and hence, the mean statistic from all three simulations is plotted here. The atmospheric model solves the fully nonhydrostatic version of the Navier-Stokes equations over an icosahedral-triangular C grid and grid size is 5 km (*59*). Vertically, the model has 90 levels with the model top at 75 km. The physics follow the ICON-Sapphire configuration in which only radiation, microphysics, and turbulence are parameterized (*60*). Parameterizations for deep and shallow convection are switched off, but in most regions, the boundary layer turbulence scheme sometimes mixes through the depth of the troposphere. Radiation scheme uses RTE-RRTMGP scheme (*61*). Microphysics uses a one-moment scheme (*62*) which simulates five hydrometeor species (rain, snow, graupel, cloud ice, and cloud water). Turbulence scheme is the Smagorinsky scheme (*63*, *64*). Land processes are simulated by the land model JSBACH including an interactive surface flux scheme and soil model (*65*, *66*). The ocean is simulated with an ocean general circulation model ICON_O_ (*67*, *68*) with a sea ice model and a biogeochemistry component from HAMOCC6 (*69*, *70*). The model grid is also an icosahedral-triangular C grid, consistent with the atmospheric grid. Before doing the coupled ocean-atmosphere simulations, the ocean is spun up by conducting an ocean-only simulation for nearly 100 years. The details of the model configurations are documented in the ICON-Sapphire model evaluation paper (*60*).

ICON_A_: Simulations from ICON configured with the same horizontal grid spacing, but forced with prescribed SSTs are used. For the current climate, we take the SSTs from the European Centre for Medium-Range Weather Forecasts Integrated Forecasting System (IFS) analysis data over 2018–2020. For simplicity, these experiments are referred to as ICON_A,2020_. Then, to analyze changes in precipitation extremes with warming, we take the SSTs from CMIP6 output of MPI-ESM 1.2-HR (*41*) for the piControl (ICON_A,1850_) and a future warming SSP585 scenario (ICON_A,2070_). Four pairs of simulations with the prescribed SSTs from the years around 1850 and 2070 were conducted. Each set of simulations was run for 6 months from 1 April to 30 September. Except that the turbulence scheme is total turbulent energy scheme (*71*), other configurations for the atmospheric model are consistent with ICON_A/O_.

Observational data are from the Integrated IMERG measurement (*72*). We compare the IMERG results with the model simulations of the current climate. IMERG has a horizontal resolution of 0.1° and a temporal resolution of half an hour. It integrates multiple satellites and uses an algorithm that incorporates gauge data over land. To compare with the models, we show the results calculated from IMERG for the same years (2018–2020) as the SSTs were taken for ICON_A,2020_ simulations. Results from IMERG computed over this period are labeled as IMERG_2020_. In addition, for the correlation plots, we calculate the IMERG results for the 20-year means over 2001–2020 to show the observed uncertainties from interannual variations. The model data are regridded to 0.1° latitude-longitude grid to match the IMERG resolution. We also test the sensitivity of the relationship between organization and precipitation extremes to resolution by regridding the model and observational data to coarser resolutions (0.2°, 0.5°, and 1°).

When comparing the model simulations of the current climate with the observations, the sample size of ICON_A_ simulation data (18 months) is quite different from those of ICON_A/O_ (33 months) and observations (36 months). But this should not be a major issue as the main results from ICON_A/O_ and ICON_A_ are very similar despite the differences in the simulation configurations and sampling size.

Daily precipitation extremes over the tropics (30°N to 30°S, land and ocean) are analyzed in this study. Precipitation extremes are determined as extreme percentiles, which are defined as the mean daily intensity over the regions where the daily precipitation amount exceeds a particular percentile of the precipitation distribution over the entire tropical domain for the same day. In particular, we focus on daily precipitation extremes at the 95th (*P*_95_), 99th (*P*_99_), and 99.9th (*P*_99.9_) percentiles, which as a whole are referred to as *P_n_*. When investigating the future changes, we also look at the daily precipitation extremes over the tropical oceans (*P*_o,*n*_). We compute all the precipitation-related variables on a daily basis. For each variable, we get one data point per day, therefore obtaining daily time series for approximately 1 year of data for ICON_A/O_ and 6 months for ICON_A_.

To better understand precipitation and its distribution, we also quantify the total area of precipitation based on the absolute daily precipitation amounts. These include the dry area (*A*_0_: *P* < 1 mm day^−1^), the light-rain area (*A*_10_: *P* > 1 mm day^−1^ and *P* < 10 mm day^−1^), the moderate-rain area (*A*_20_: *P* > 10 mm day^−1^ and *P* < 20 mm day^−1^), and the heavy-rain area (*A*_50_: *P* > 50 mm day^−1^).

The degree of convective organization is measured with a clustering organization metric: *I*_org_ (*29*). It is a nondimensionalized variable which measures the nearest-neighbor distance between deep convective centroids and then compares the cumulative density function of these nearest-neighbor distances against the distribution assuming that the clusters (with the same number) are randomly distributed. In this study, we calculate the nearest-neighbor distances between the centers of mass (centroids) of the convective clusters. We also test the “local minimum” method (*33*, *34*) and identify the convective centroids by the “local maximum” of daily mean precipitation, the main results are consistent, and *I*_org_ is not very sensitive to how convective centroids are identified (center of mass or local maximum of daily mean precipitation). *I*_org_ varies between 0 and 1. Larger *I*_org_ indicates a more organized state. In this study, the convective regions are identified as grids with daily precipitation amount exceeding 95th quantile over the entire tropical domain (30°N to 30°S, land and ocean) on a given day. Note that we apply a relative threshold of precipitation to characterize convective organization because daily precipitation statistics tend to change with warming. Using 95th quantile as the threshold to identify convection has the advantage that the total number of convective grids on each day is always fixed (5% of the domain). This allows the metric to be applied in different climates when the mean state changes. In principle, this should not benefit the relationship between *I*_org_ and precipitation extremes, as precipitation data are only used to identify the convective grids, and afterward, the data become a binary field which does not contain precipitation information. Other thresholds (90th, 97th, and 99th quantiles of daily precipitation) are also applied to test the robustness, and the results are qualitatively consistent (not shown). Apart from using daily precipitation data to compute *I*_org_, we also test parts of the results by applying outgoing longwave radiation (OLR) as the variable to measure organization (*I*_org,olr_). Following the method introduced in the RCEMIP protocol paper (*22*), we compute *I*_org,olr_ using OLR at 3-hourly intervals and identify convective grids by instantaneous OLR values less than 173 W m^−2^. Then, we average the instantaneous *I*_org,olr_ by day to obtain the daily mean *I*_org,olr_. Consistent with our expectation, the identified convective clusters from OLR are less numerous in number. As OLR is sensitive to high clouds, it can miss out on smaller-scale organization structure occurring underneath the high cloud decks. Nevertheless, the main results are qualitatively consistent, e.g., both the absolute values and changes of *I*_org,olr_ and *I*_org_ are linearly correlated (fig. S13, A and C), *I*_org,olr_ increases with warming (fig. S13B), and changes in precipitation extremes are closely linked to changes in *I*_org,olr_ (fig. S13D).

We first look for the convective grids (which are the top 5% of the grids with the highest daily precipitation amount). The identified convective grids are marked as 1, and the remaining domain (nonconvective) are marked as 0. Thus, the fields become binary. Two convective grids belong to one convective cluster if they share a boundary (at least four grid points connected). We count the total number of the convective clusters as *N*. As the total convective grids (*N*_tot_) are fixed (5% of the domain), the effective radius of the convective clusters which we call size (*S*) can be obtained as

$$S=\sqrt{\frac{\Delta x*\Delta x}{\pi }\frac{N_{\text{tot}}}{N}}$$ (1)

where Δ*x* is the horizontal grid spacing of the data. *N* or *S* can be considered as an additional metric to complement *I*_org_, as they focus on different characteristics of organization. While *I*_org_ features spatial clustering, *N* and *S* are not affected by that. Consistent with how precipitation-related variables are calculated, we also obtain daily time series of organization-related variables.

To illustrate the evolution of convective organization and how the organization metrics capture the changes, we show the snapshots of daily precipitation and identified convective objects for five consecutive days over a tropical ocean area simulated by ICON_A/O_ (fig. S14). The degree of convective organization evolves from initially more scattered convection to finally a more organized state. With increased organization, *N* decreases substantially while *S* increases. Meanwhile, *I*_org_ increases monotonically from day 1 to day 5. All the metrics are able to capture such an increase in the degree of mesoscale organization.

A first inspection of these, data show that daily mean precipitation ( $\bar{P}$ ), daily extreme precipitation (*P_n_*), and the degree of mesoscale organization are inter-related, and *P_n_* tends to increase with both $\bar{P}$ and *I*_org_. Such a covariability is likely driven by seasonal changes in the large-scale circulation. To remove the large-scale signal, a deseasonalized statistic (signified with δ) for each variable is derived by subtracting the corresponding 30-day running-averaging values. Now, we have the deseasonalized time series of all the variables considered. When estimating the correlation coefficient (*R*) from these daily time series data, we want to make sure that the calculated *R* between δ*I*_org_ and δ*P*_*n*_ does not come from their covariability with $\delta \bar{P}$ . Therefore we divide the data into several bins by $\delta \bar{P}$ : Each bin has a small variation in $\delta \bar{P}$ . By doing so, $\delta \bar{P}$ is constrained to be almost uncorrelated with δ*I*_org_. We then compute *R* between δ*I*_org_ and δ*P*_*n*_ in each bin, and the averaged *R* across all bins is used in the analysis. The same analysis is applied to calculate R between δ*I*_org_ and other variables. In addition, to understand the link between convective organization and the dynamics of extreme precipitation, we analyze the vertical velocity at 500 hPa (ω_500_) corresponding to the extreme precipitation events. Because of the lack of vertical velocity data in observations, this analysis is only carried out with the ICON_A/O_ data.

For changes in precipitation extremes with warming, the analyses are based on simulation output of ICON_A,1850/2070_. The way to compute the future changes is more straightforward because when calculating the changes between two periods, the data are naturally deseasonalized. Daily precipitation extremes and organization metrics are first computed and averaged in time (monthly from April to September). Thus, we obtain daily precipitation extremes and organization for each month. In total, we have 24 cases (including 6 months from four pairs of experiments) for each variable. These 24 cases are considered as simulation ensembles. Changes in precipitation and organization in a warming scenario can be directly computed using the mean statistics from each month in 2070 against them in 1850. For precipitation (including mean and extremes), it is calculated as a scaling ratio (α) following

$$P_{2070}=P_{1850}{\left(1+0.01\alpha \right)}^{\frac{\Delta T}{1K}}$$ (2)

where *P*_1850_ and *P*_2070_ are daily precipitation amounts in 1850 and 2070, respectively. The exponent is nondimensionalized by 1 K, and Δ*T* is the mean surface temperature increase in 2070 relative to 1850. Equation 2 describes a relationship that precipitation changes exponentially with temperature increase.

To help understand changes in daily precipitation extremes, we further decompose *P_n_* into precipitation intensity or magnitude (*M_n_*) and precipitation duration (*D_n_*) following

$$\begin{array}{c} P_{n}=\frac{P_{n}}{D_{n}}*D_{n}\\ =M_{n}*D_{n} \end{array}$$ (3)

Using the half-hourly precipitation data, we focus on the grids of *P_n_* and then count the total rainy hours within 1-day period. The rainy hours are identified as the time when the half-hourly precipitation intensity > 1mm hour^−1^. Then, *D_n_* is the total rainy hours of the day, and *M_n_* is diagnosed by dividing *P_n_* by *D_n_* which is the mean precipitation rate averaged only over the rainy period. Therefore, changes in daily precipitation extremes (Δ*P*_*n*_) is decomposed into changes in the precipitation intensity (Δ*M*_*n*_) versus duration (Δ*D*_*n*_).
