# Supplementary material for: Intensification of daily tropical precipitation extremes from more organized convection
Source: Sci Adv. 2024 Feb 23;10(8):eadj6801. doi: 10.1126/sciadv.adj6801 (PMC10889435; doi:10.1126/sciadv.adj6801)
Supplement: Supplementary file 1 — Figs. S1 to S14 [file sciadv.adj6801_sm.pdf]

Science Advances
AAAS

Supplementary Materials for

Intensification of daily tropical precipitation extremes from more organized convection

Jiawei Bao *et al.*

Corresponding author: Jiawei Bao, jiawei.bao@mpimet.mpg.de

Sci. Adv. **10**, eadj6801 (2024)
DOI: 10.1126/sciadv.adj6801

This PDF file includes:

Figs. S1 to S14

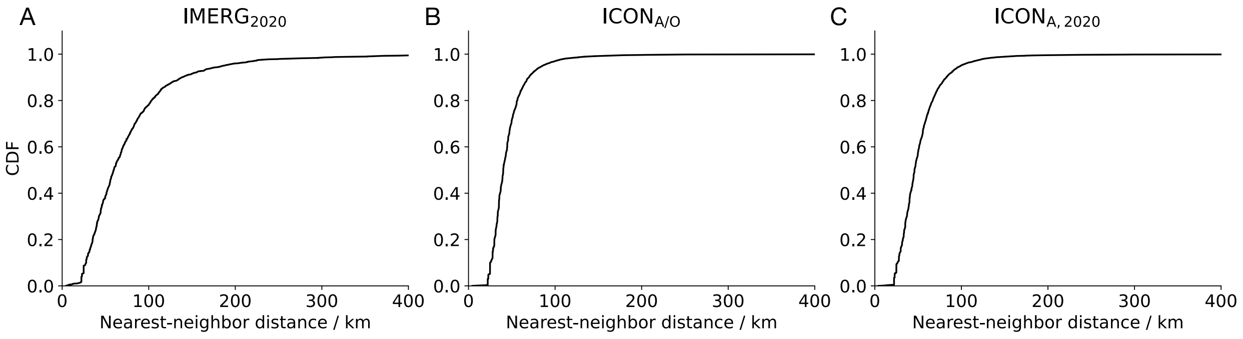

Fig. S1.

Scales of convective organization measured by I_{org}. Cumulative distribution functions (CDF) of nearest neighbor distances of convective clusters for one-day snapshots over the whole tropics from IMERG_{2020} (**A**), $\text{ICON}_{A/O}$ (**B**), $\text{ICON}_{A,2020}$ (**C**).

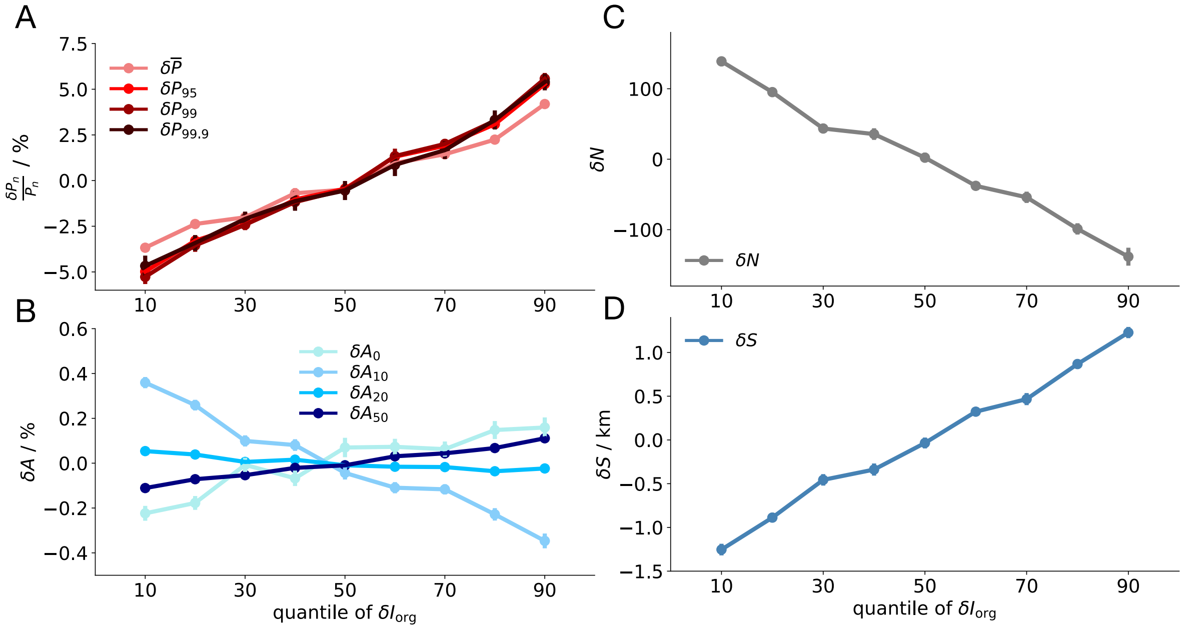

Fig. S2.

Observed precipitation-related and organization-related statistics sorted by organization. The deseasonalized precipitation amounts (δP_n normalized by the mean P_n, **A**), the area of precipitation(δA, **B**), Number (δN, **C**) and Size (δS, **D**) of convective clusters as a function of mean quantiles of the deseasonalized convective organization δI_{org} (e.g. 50th quantile corresponds to the averaged values of δI_{org} that is between 45 and 55th quantiles). The results are shown for 20 years of IMERG with The error bars shows the standard errors from yearly variations.

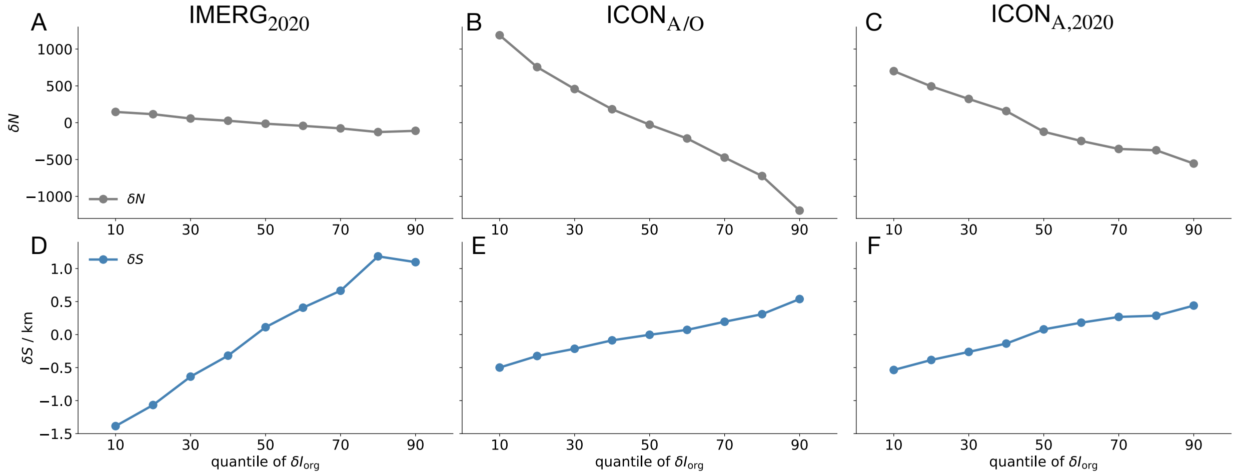

Fig. S3.

Number and Size of convective clusters sorted by organization. The deseasonalized Number (δN, **A-C**) and Size (δS, **D-F**) of convective clusters as a function of mean quantiles of the deseasonalized convective organization δI_{org} (e.g. 50th quantile corresponds to the averaged values of δI_{org} that is between 45 and 55th quantiles). The results are shown for IMERG_{2020} (**A**,**D**), $\mathrm{ICON}_{\mathrm{A/O}}$ (**B**,**E**) and $\mathrm{ICON}_{\mathrm{A,2020}}$ (**C**,**F**).

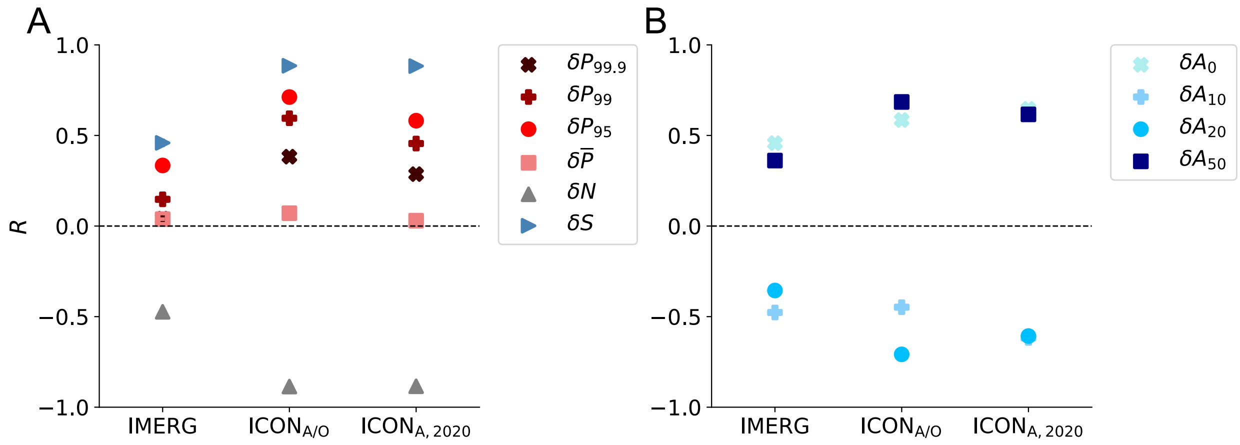

Fig. S4.

Correlation coefficients between convective organization and daily precipitation extremes. Correlation coefficients (R) between deseasonalied I_{org} and precipitation or organization variables from IMERG for the 20-year means, ICON$_{A/O}$, ICON$_{A}$. (**A**). R between δI_{org} and daily extreme precipitation ($\delta P_{99.9}$, δP_{99} and δP_{95}), mean precipitation ($\delta \overline{P}$), total number (δN) and mean size (δS) of convective objects. (**B**). R between δI_{org} and the dry area (δA_0), the light-rain area (δA_{10}), the moderate-rain area (δA_{20}) and the heavy-rain area (δA_{50}). R is calculated by conditioning on $\delta \overline{P}$. Error bars (±one standard error of R calculated by year) are added on top of the 20-year means of R from IMERG.

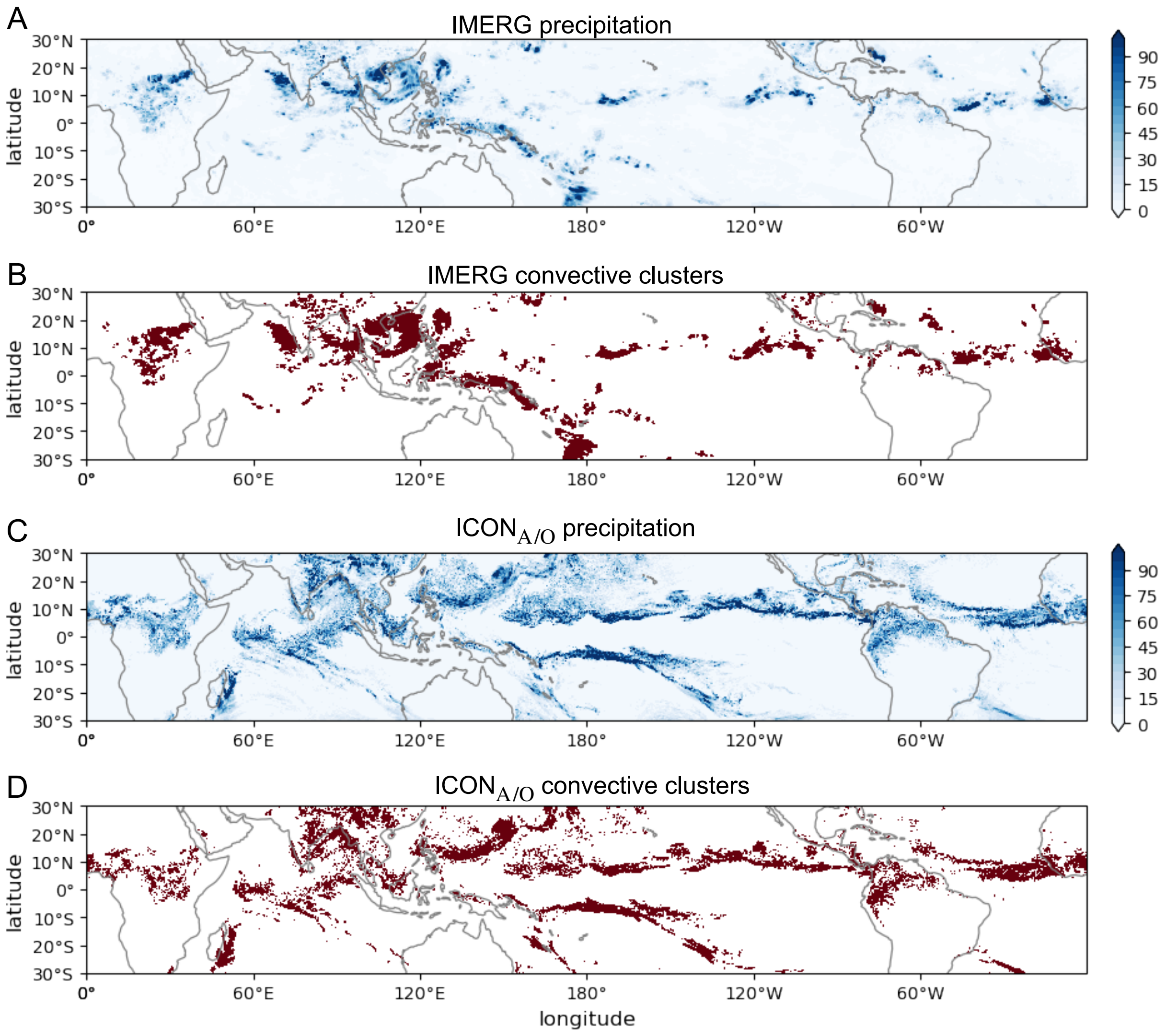

Fig. S5.

Spatial distribution of precipitation and convective clusters. Snapshots of daily precipitation distribution (mm day^{-1}, **A** and **C**) and identified convective clusters (**B** and **D**) over the tropics (30°N-30°S) from IMERG and ICON$_{A/O}$ simulations.

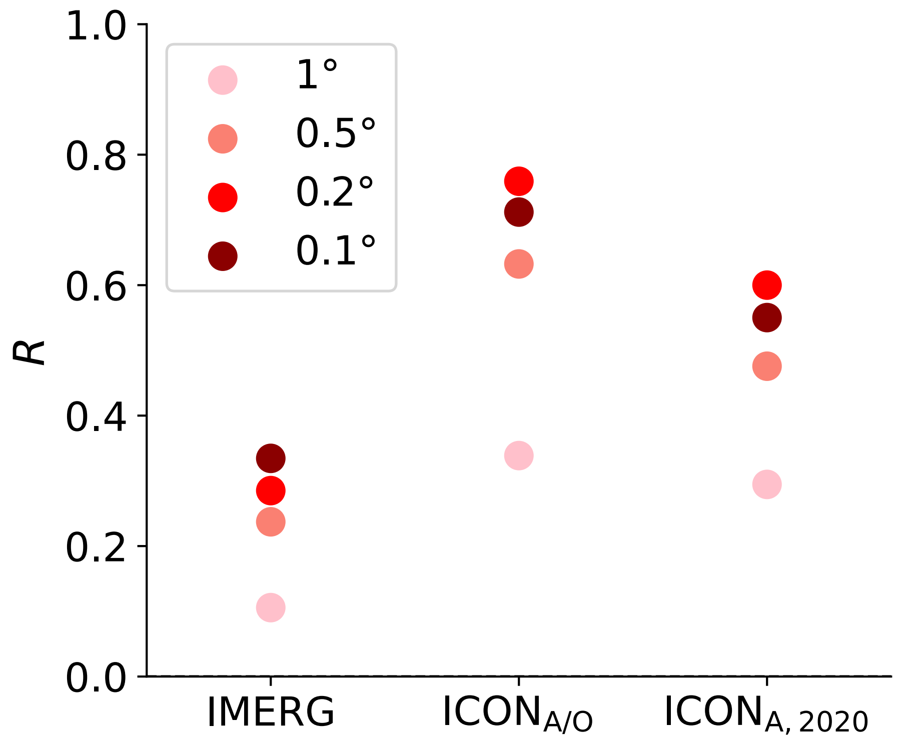

Fig. S6.

Resolution dependence of the relationship between daily precipitation extremes and organization. Correlation coefficients (R) between δI_{org} and δP_{95} from IMERG for the 20-year means, ICON$_{A/O}$, ICON$_{A}$. The data are regridded to different horizontal resolutions (1˚, 0.5˚, 0.2 ˚, 0.1˚). R is calculated by conditioning on $\delta \overline{P}$ with the data that are deseasonalized. Error bars (± one standard error of R calculated by year) are added on top of the 20-year means of R from IMERG.

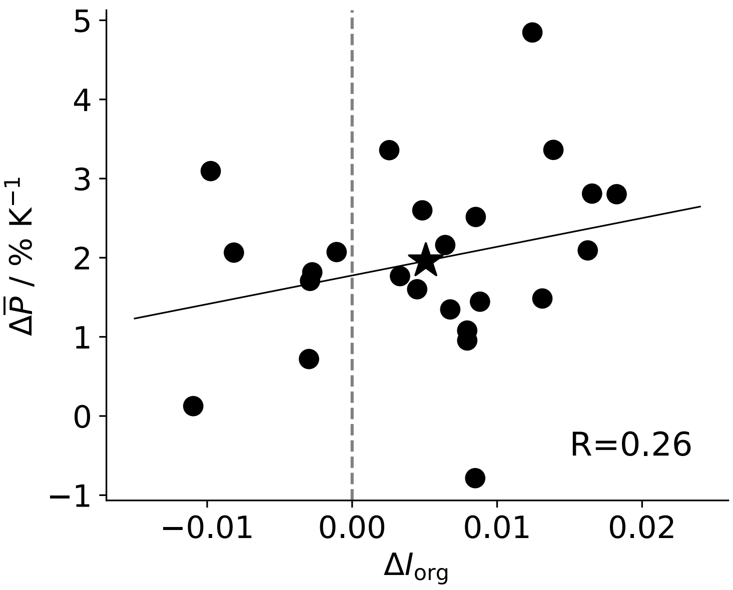

Fig. S7.

Changes in organization vs. changes in mean precipitation. Changes in daily mean precipitation ($\Delta\overline{P}$) vs. changes in ΔI_{org}. The changes are computed between 2070 and 1850, and are normalized by the mean surface temperature increase. Each dot represents the daily change averaged in one month. Pentagrams are the mean values of all months across all ensemble members. The results are obtained from $\mathrm{ICON_A}$.

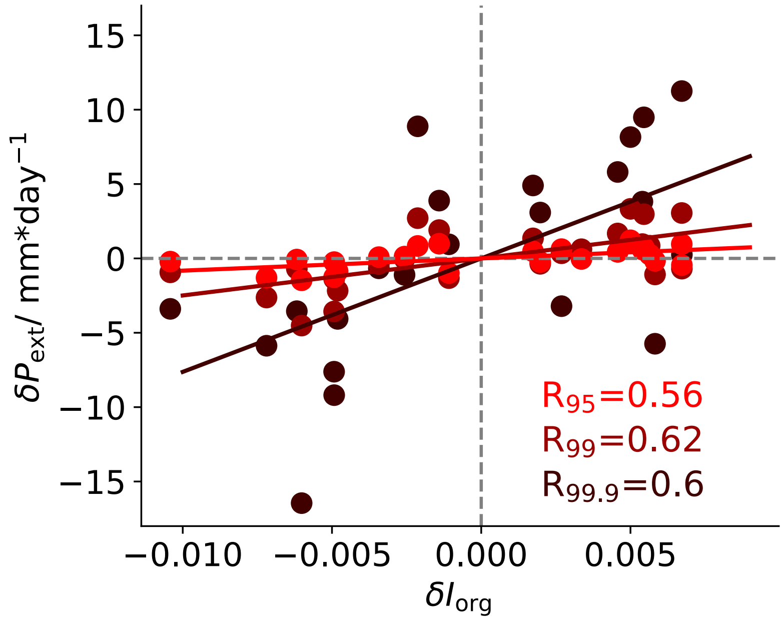

Fig. S8.

Monthly variability in mean organization vs. monthly variability in daily extreme precipitation. Deseasonalized monthly mean convective organization (δI_{org}) vs monthly mean daily precipitation extremes (δP_n) (mm day^{-1}) in 1850. Monthly averaging is performed before deseasonalization which subtracts the mean monthly averaged variables across the simulations. Each dot represents the mean values in one month. The results are obtained from $ICON_A$.

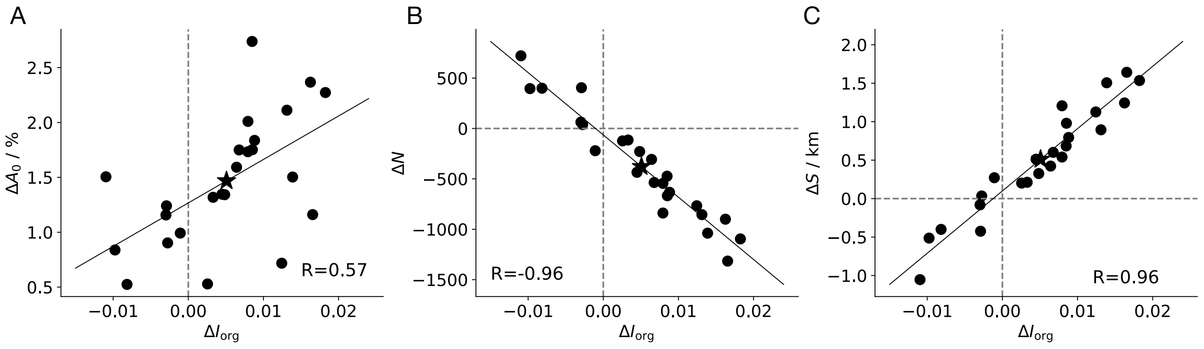

Fig. S9.

Changes in dry area and organization-related statistics vs. changes in organization with warming. Changes in the dry area (ΔA_0, **A**), number (ΔN, **B**) and (ΔS, **C**) vs. changes inΔI_{org}. The changes are computed between 2070 and 1850. Each dot represents the daily change averaged in one month. Pentagrams are the mean values of all months. The results are obtained from $\mathrm{ICON_A}$.

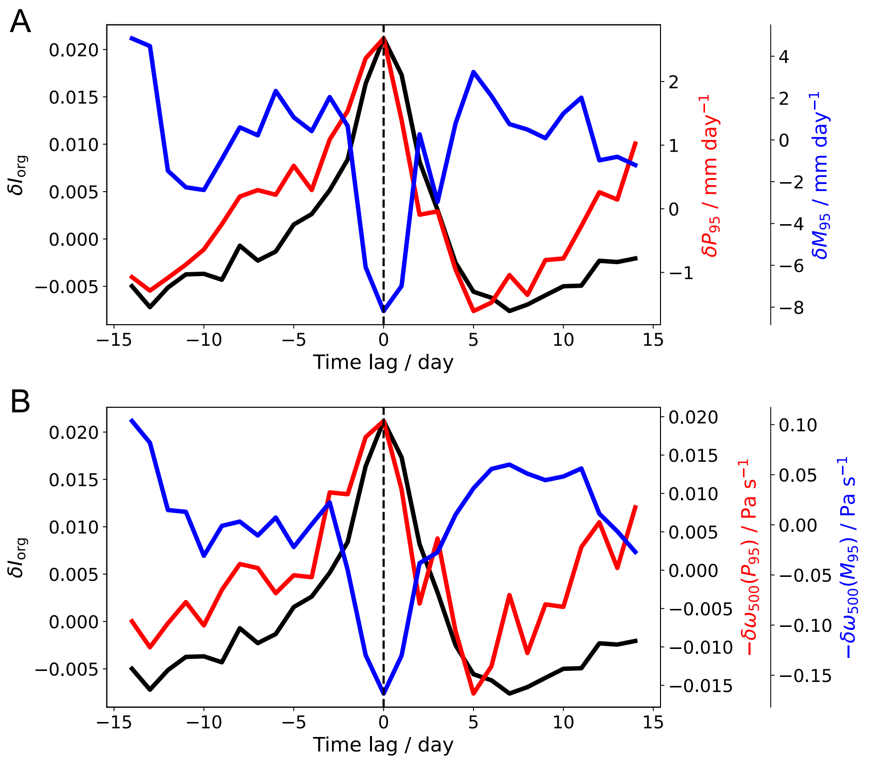

Fig. S10.

Impact of organization on the dynamics of precipitation extremes. (**A**). Time evolution of deseasonalized convective organization (δI_{org}), 95th percentile of daily precipitation amount (δP_{95}), and the intensity of precipitation over the rainy hours (δM_{95}) during the composite peak organization events. (**B**). Time evolution of deseasonalized convective organization (δI_{org}), daily mean vertical velocity at 500 hPa corresponding to 95th percentile of daily extreme precipitation accumulations ($\delta\omega_{500}(P_{95})$) and hourly mean vertical velocity at 500 hPa corresponding to the intensity of precipitation over the precipitating hours ($\delta\omega_{500}(M_{95})$) during the composite peak organization events. The details of the plot are similar to Figure 1. Vertical velocity in b is plotted with a minus sign (positive value indicates an increase in updraft velocity). The results are shown for one of the $ICON_{A/O}$ simulations.

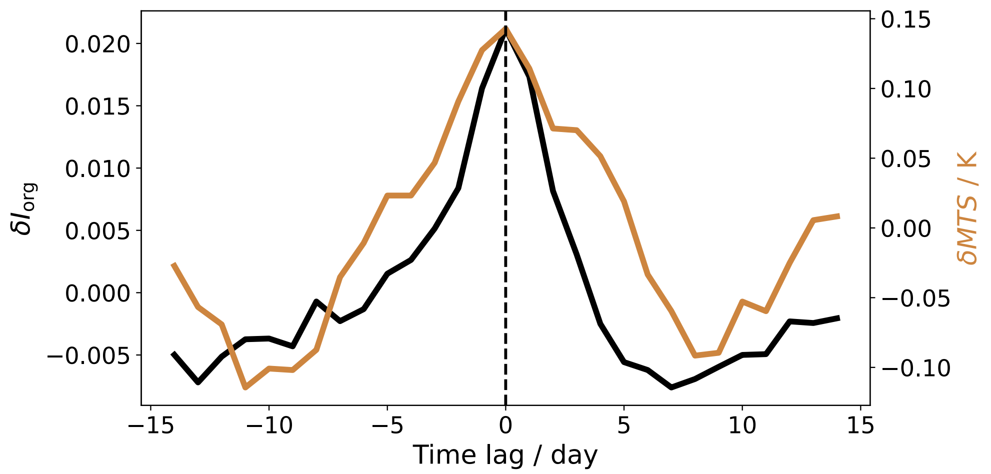

Fig. S11.

Convective organization and the tropospheric stability. Time evolution of deseasonalized convective organization (δI_{org}) and mid-tropospheric stability (δMTS) during the composite peak organization events. MTS is the difference between the potential temperature of the mid-troposphere (500 hPa) and near the surface (1000 hPa). The details of the plot are similar to Figure 1. The results are shown for one of the $\text{ICON}_{\text{A/O}}$ simulations.

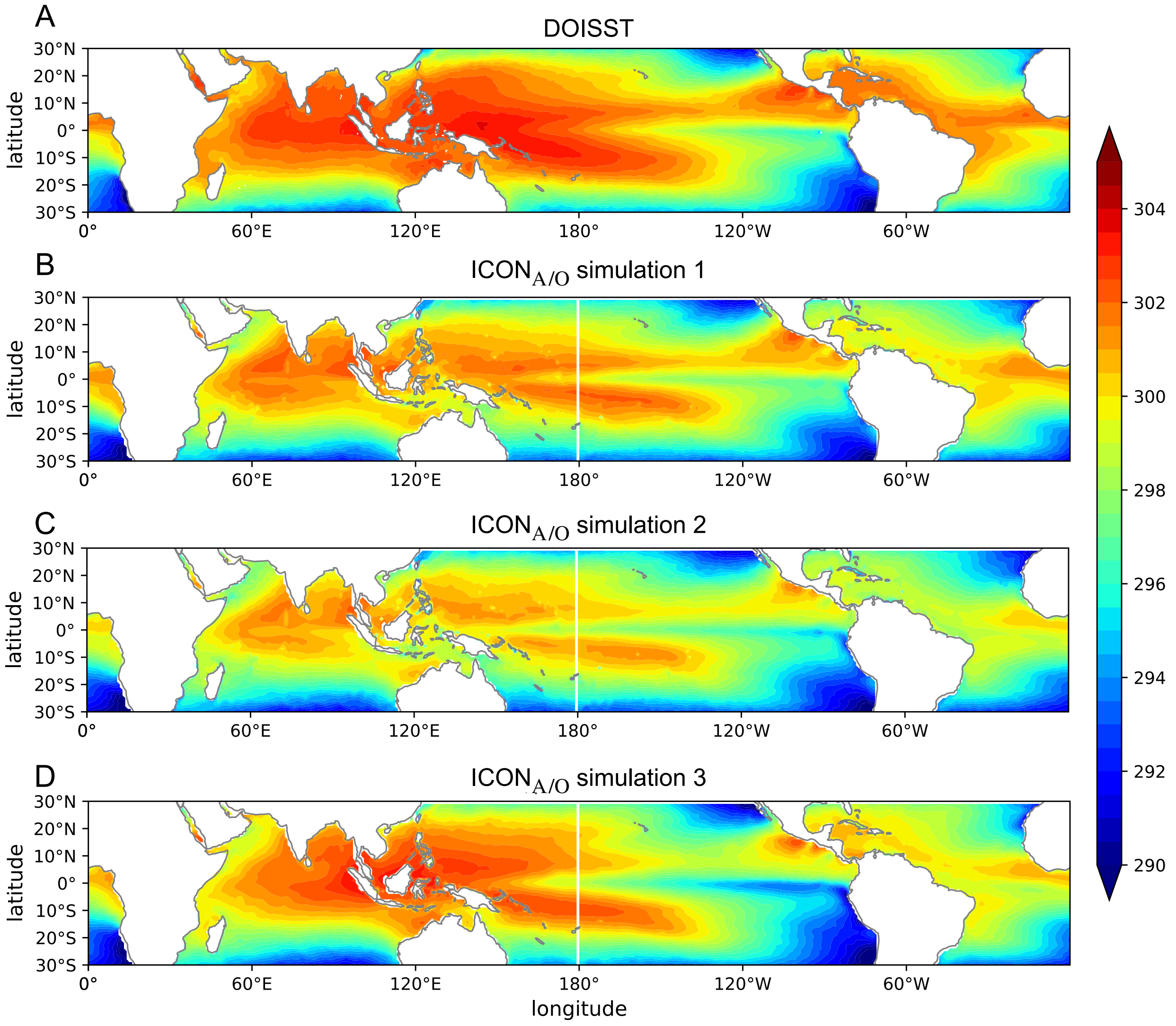

Fig. S12.

Mean sea surface temperature (K) distributions averaged over 2020 from OISST (**A**) and three ICON$_{A/O}$ simulations (**B-D**).

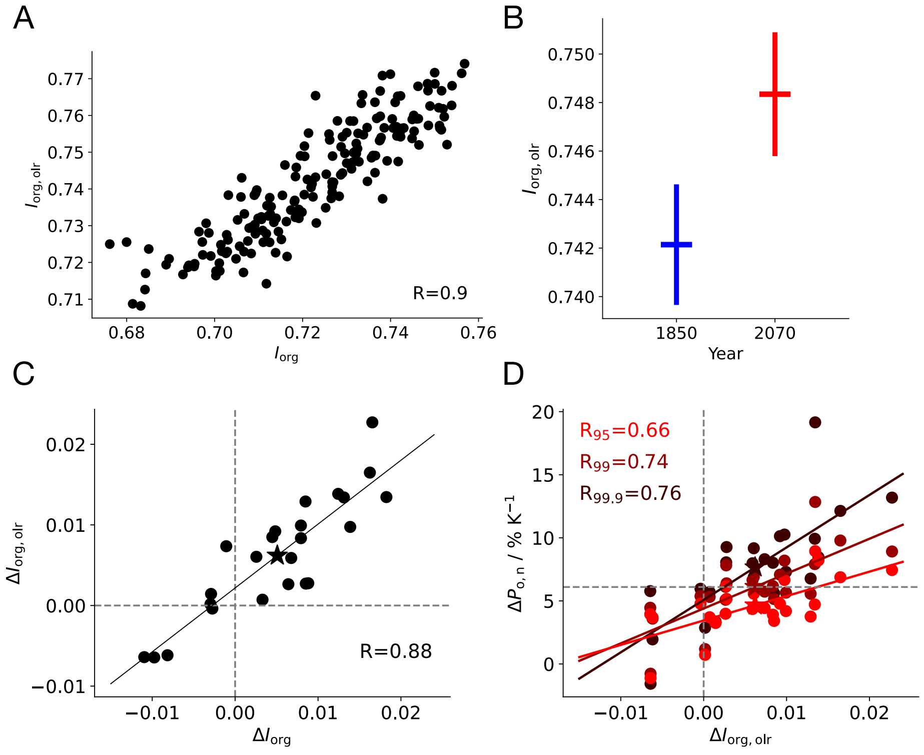

Fig. S13.

(**A**) Daily variations of organization calculated using daily precipitation data (I_{org}) vs. daily variations of organization calculated using instantaneous outgoing longwave radiation ($I_{org,olr}$) from one of the ICON$_A$ simulations. (**B**) Mean $I_{org,olr}$ in 1850 vs. 2070. The error bars show standard errors from monthly variations. (**C**) Changes in monthly mean organization calculated using daily precipitation (ΔI_{org}) vs. changes in organization calculated using instantaneous outgoing longwave radiation ($\Delta I_{org,olr}$). (**D**). Changes in daily precipitation extremes over the ocean ($\Delta P_{o,n}$) vs. changes in $\Delta I_{org,olr}$. The changes are computed between 2070 and 1850 using simulations by ICON$_A$. Each dot represents the daily change averaged in one month. The details of Figure (**D**) are similar to Figure 3**D**.

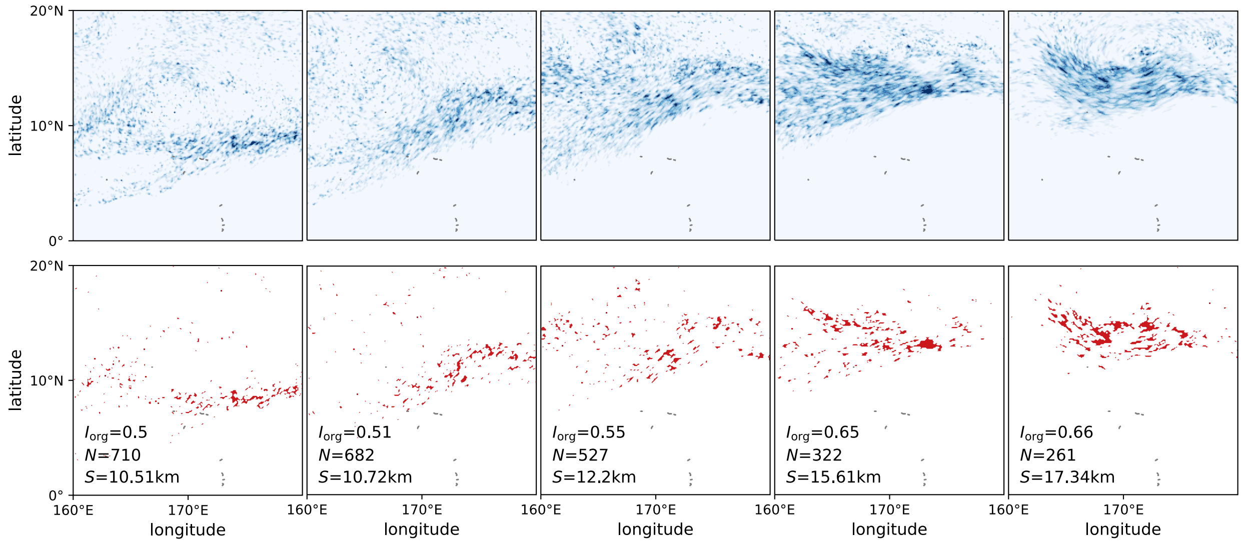

Fig. S14.

Snapshots of daily precipitation distribution and identified convective clusters for 5 consecutive days over a tropical ocean area (0˚-20˚S, 160˚E-180˚E) as simulated by $ICON_{A/O}$. Values of convective organization metrics (I_{org}, N and S) calculated over this smaller domain are also shown.
